# Near-infrared light stimulation regulates neural oscillation and memory behavior of mice with Alzheimer’s disease

**DOI:** 10.3389/fnins.2024.1417178

**Published:** 2024-10-21

**Authors:** Song Zhang, Xiaopeng Wang, Honglei Jiao

**Affiliations:** Department of Neurology, The Second Hospital of Hebei Medical University, Shijiazhuang, China

**Keywords:** Alzheimer’s disease, photobiomodulation, electroencephalogram, neural oscillations, near-infrared light stimulation

## Abstract

Photobiomodulation (PBM) is a non-invasive neuromodulation technique for the brain. Low-intensity near-infrared light (1–500 mw) has demonstrated the ability to improve memory in Alzheimer’s disease (AD) model mice, suggesting its potential for AD treatment. However, the impact of PBM on neural oscillations in the hippocampal region affected by AD remains unknown. In this study, AD model mice were subjected to PBM for 60 days and then tested using novel object recognition behavior (NOR) experiments. During behavioral experiments, local field potential signals (LFP) of the mice was recorded using a single electrode in the CA1 region to analyze memory ability and neural oscillation characteristics. The results revealed that mice stimulated with PBM exhibited significantly higher new object differentiation indices compared to the Sham group (*p* < 0.01). Furthermore, PBM stimulation led to a significant increase in relative power and sample entropy of theta and gamma bands (*p* < 0.01). The coupling intensities of θ-low-γ and θ-high-γ were also significantly higher in the PBM group compared to the Sham group (*p* < 0.01). In conclusion, these findings suggest that PBM may improve memory ability in AD mice through regulation of neural oscillation characteristics, providing a theoretical basis for utilizing PBM as a treatment modality for Alzheimer’s disease.

## 1 Introduction

Alzheimer’s disease (AD) stands as the predominant form of dementia (Vermunt et al., 2019). It inflicts damage on patients’ memory and cognitive abilities, profoundly affecting their daily life and reducing overall quality of life. Present AD treatments primarily rely on drug therapy, which often entails various side effects, including dosage-related issues (Tatulian, 2022). Photobiomodulation (PBM) is a non-invasive neuroregulation technique targeting the brain. It utilizes low-intensity near-infrared light (1 − 500 mw) known to trigger beneficial biological processes within tissues and demonstrates efficacy in treating various diseases (Disner et al., 2016; Huisa et al., 2013; Meng et al., 2013; Tatmatsu-Rocha et al., 2018; Tao et al., 2021; Zhao et al., 2022).

Numerous studies have affirmed the positive therapeutic and protective effects of PBM on physiological changes induced by AD (Tao et al., 2021; Purushothuman et al., 2014; Grillo et al., 2013; Farfara et al., 2015). PBM not only enhances memory in AD model mice (Meng et al., 2013; Tao et al., 2021; Grillo et al., 2013) but also shows promise in reducing Aβ burden and phosphorylated tau protein levels in plasma (De Taboada et al., 2011), cerebrospinal fluid (De Taboada et al., 2011), and the brain (Purushothuman et al., 2015), highlighting its significant therapeutic potential for AD. Despite these physiological insights, numerous studies have revealed that AD induces dramatic alterations in neural oscillations within the hippocampus (Jafari et al., 2020). However, limited research has delved into the impact of PBM on neural oscillations in AD models. This study primarily investigated the regulatory effects of PBM on neural oscillations in the CA1 region of AD model mice, with a focus on deciphering the neuroregulatory mechanisms of PBM on AD from an electrophysiological perspective.

This study applied prolonged PBM stimulation to AD model mice, recorded local field potential (LFP) signals in the CA1 region utilizing single electrodes during novel object recognition behavior (NOR) experiments, and analyzed the memory capabilities and neural oscillation characteristics of mice. The goal was to provide insights into the neural mechanisms underlying PBM treatment in AD model mice. Our findings offer valuable guidance for the development and clinical application of PBM.

## 2 Materials and methods

### 2.1 Animals

**FIGURE 1:**
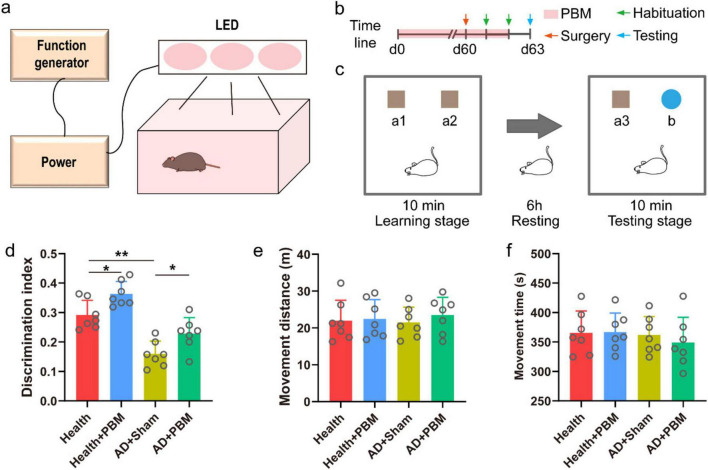
**(a)** Schematic diagram of PBM device. **(b)** Timeline diagram. **(c)** Schematic diagram of NOR experiment. **(d)** The result of discrimination index of new object in NOR experiment test stage. **(e)** The movement distance of mice in the test stage. **(f)** The movement time of mice in the test stage.

A total of 21 C57BL/6 mice and 395 × FAD mice (4 months old) (male) were utilized in the study. The overall timeline of the experiment is depicted in Figure 1b. Following 60 days of PBM stimulation, electrodes were implanted, and the mice were acclimated to the environment over the subsequent two days. NOR experiments and electroencephalogram (EEG) signal collection were carried out on the 63rd day. In the experiment, we adopted the principle of random grouping and randomly assigned the AD mice to the treatment group and the control group. To verify the reliability of the experiment, AD mice of the same age were randomly selected and randomly divided into two groups. The results showed that there was no significant difference in PAC between the two groups, proving that the random grouping was reliable (Supplementary Figure 1). All mice were individually housed in standard cages with free access to food and water in a controlled environment with a 12-h light and 12-h dark cycle and consistent temperature and humidity. All procedures were performed in accordance with the guidelines of the Animal Ethics Committee of the second hospital of Hebei Medical University. And the approval letter number is 2023-AE-130.

### 2.2 Surgery

On day 60 of PBM stimulation, single channel nickel-chromium electrodes (Beijing Creation Technologies Co., Ltd.) were implanted in the CA1 region of the mice following established procedures (Wang et al., 2021). Briefly, after removing the fur above the mouse’s skull and cutting the skin, the subcutaneous tissue was excised, and a circular portion of the skull with a diameter of ∼1.5 mm was removed for electrode implantation with the center coordinates being anteroposterior distance of −2 mm, mediolateral distance of 1.5 mm, and dorsoventral distance of −1.5 mm. The tissue section of the electrode implantation location is shown in Supplementary Figure 2. Two holes were drilled in the nasal bone to affix the ground and reference electrodes. Finally, all electrodes were securely fixed to the skull using glue and dental cement (Super-Bond C&B). The entire surgery was conducted under anesthesia using 4% isoflurane for induction and 1.5–2% isoflurane for surgery with ∼0.5 L/min O2.

### 2.3 PBM protocol

The PBM device, as illustrated in Figure 1a, was constructed based on previous studies (Tao et al., 2021), comprising a chamber and a top cover assembly with an array of light-emitting diodes (LEDs). The LED array had an average power of 900 mW, an average power density of 25 mW/cm2, a total radiant exposure of 4.5 J/cm2, and a light pulse frequency of 10 Hz at 50% duty cycle. A cooling fan was incorporated to mitigate the thermal effects of the LED array. Mice in the AD++PBM group received 6 min of exposure every day at 7 pm for 60 consecutive days, with the light device emitting at a wavelength of 1070 ± 50 nm. During the treatment, mice were allowed to move, explore, and rest. Mice in the AD+Sham group and the health group underwent a similar treatment regime, except that the light device remained turned off.

### 2.4 NOR experiments

NOR experiments were performed as previously reported (Taglialatela et al., 2009). Initially, mice were trained for two days, during which they freely explored an alcohol-wiped experimental box (45 cm × 45 cm × 30 cm) for 15 min. The formal experiment, outlined in Figure 1c, commenced with a 10-min free exploration phase in an alcohol-disinfected experimental box. Two identical brown cubes (a1 and a2) with sizes of 5 cm × 5 cm × 5 cm were placed equidistantly in the center. After a 6-h rest in a dedicated transfer cage, the testing phase began with exploring the experimental box for 10 min. An entirely new object (a3), identical to a1, and a randomly selected blue cylinder object b were placed in the box to replace either a1 or a2. The total time spent exploring a3 (T_familiar_) and the total time spent exploring object b (T_novel_) were recorded. The discrimination index (DI) was calculated as DI = (T_novel_ - T_familiar_) / (T_novel_ + T_familiar_). The total time and distance of mouse activity during the test phase were also recorded to assess the mice’s movement status. We refer to previous literature to calculate movement distance and time (Yuan et al., 2020). We also calculated the DI and exploration time of each group of mice in the learning phase.

### 2.5 Data acquisition

The electrical signals of mice’ brains were recorded during the 10-min test phase using a multi-channel acquisition system (Apollo, Bio-Signal Technologies: McKinney, TX, USA). The electrodes implanted in the brain were connected to this system via front amplifiers and analog-to-digital converters. The sampling rate for LFP signals was 1 kHz, while the sampling rate for spike and raw data was 30 kHz.

### 2.6 Power spectrum and relative power

The power spectral density of LFP signals was calculated using the Welch algorithm in Matlab software. The absolute power within the theta (θ, 4 − 12 Hz) and gamma (γ, 30 − 120 Hz) frequency bands was computed using the Bandpower function. Relative power was obtained by dividing the absolute power within these bands by the absolute power within the 0.5 − 200 Hz range.

### 2.7 Sample entropy

Sample entropy for the above frequency bands was calculated based on previous studies (Wang et al., 2023) using Eq. 1:


(1) $$S\,a\,m\,p\,l\,e\,E\,n\,\left(m,r,N\right)=-\ln\left[C^{m+1}\,\left(r\right)/C^{m}\,\left(r\right)\right]$$

where N is the data length, m is the vector dimension, and r is the tolerance. Each data segment’s length was set to 1000, the vector dimension was set to 2, and the tolerance was set to 0.2. After calculating the data of each segment, the average sample entropy for each group was computed using the mean method.

### 2.8 Phase-amplitude coupling

The LFP signals were segmented into 60-s intervals, and phase-amplitude coupling indices (PACI) at the phases of delta (δ, 1 − 4 Hz) − low gamma (low γ, 30 − 80 Hz), delta − high gamma (high γ, 80 − 120 Hz), θ − low γ, and θ − high γ were calculated as previously reported (Voytek et al., 2013). The average PACI for each segment was determined. PACI values positively correlated with phase-amplitude coupling strength, as indicated by Eq.2:


(2) $$P\,A\,C\,I=\left|\frac{1}{K}\,\sum_{k=1}^{K-1}\exp\left(i\,\left(\phi_{l}\,\left[k\right]-\phi_{h}\,\left[k\right]\right)\right)\right|$$

where *k* is the time index, *ϕ_*l*_* is the phase of low-frequency oscillations, and *ϕ_*h*_* is the phase of high-frequency oscillations after bandpass filtering to extract the low-frequency component.

### 2.9 Statistical analysis

All data were presented as mean ± standard deviation. Statistical analysis employed a one-way analysis of variance (ANOVA), with a significance level set at *P* ≤ 0.05. GraphPad Prism 8 was used for all statistical analyses.

## 3 Results

### 3.1 PBM improves the working memory of AD mice

We conducted a NOR behavior test to assess the DI, activity time, and travel distance during the testing phase. Initially, we compared the DI of AD mice with that of normal mice during the testing phase and observed a significant decrease in the DI percentage of mice in the AD+Sham group compared to mice in the health group (Figure 1d, 0.159 ± 0.044 *vs.* 0.292 ± 0.050, *p* < 0.01). This indicates a memory impairment in AD mice in the experiment. However, after prolonged PBM stimulation, mice in the AD+PBM group showed a significantly increased DI during the testing phase compared to mice in the AD+Sham group (0.229 ± 0.054 *vs.* 0.159 ± 0.044, *p* < 0.05). The results demonstrated that 62 days of PBM stimulation enhanced AD mice’ learning and memory abilities. To evaluate whether the movement status of AD mice was influenced by PBM, thereby indirectly affecting the degree of memory for objects, we measured the activity time and travel distance during the exploration phase (Figures 1e, f). The results showed that the movement status of AD mice was not significantly affected by PBM. In conclusion, PBM stimulation for 62 days significantly improved AD mice’s learning and memory capabilities, but not their movement status. We also verified the behavioral results of healthy mice after PBM stimulation. The results showed that the DI after PBM was significantly higher than that of unstimulated healthy mice (0.29 ± 0.050 vs. 0.36 ± 0.042, *p* < 0.05) (Figure 1d), the above results indicate that PBM does not specifically treat AD mice. In order to rule out whether mice have specific choices for recognition targets, we counted the exploration time and DI during the training phase and there was no significant difference (Supplementary Figure 3). It proved that mice were basically interested in each recognition target. We verified the behavioral results of normal mice after PBM stimulation, and the change trend was similar to that of AD mice. DI after stimulation was significantly higher than that of unstimulated mice (0.292 ± 0.050 vs. 0.363 ± 0.042, *p* < 0.05) (Figure 1d), indicating that PBM had no specific effect on AD mice.

### 3.2 PBM modulates theta and gamma band features in the CA1 region of AD mice

**FIGURE 2:**
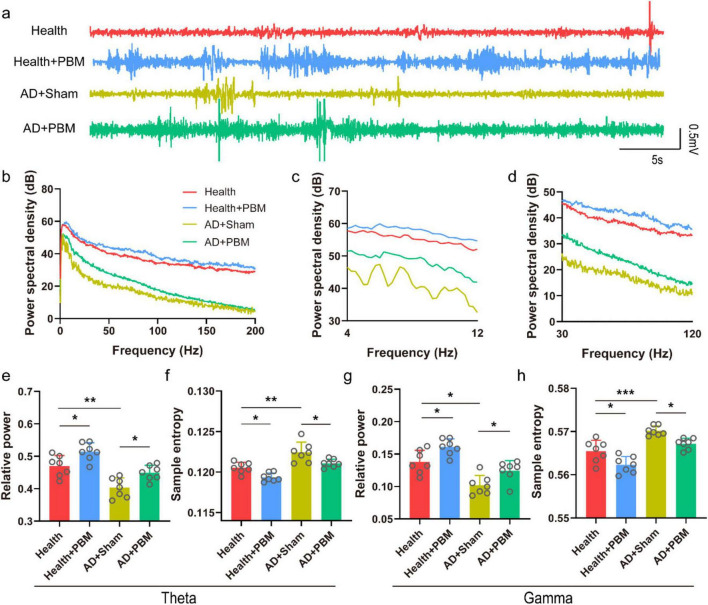
**(a)** Typical LFP diagram of each group of mice (randomly selected data for 1-min, non-average data). **(b)** Corresponding to the 0–200 Hz power spectrum of each LFP data in panel **(a)**. **(c)** Power spectrum of the theta band in panel **(b)**. **(d)** Power spectrum of the gamma band in panel **(b)**. **(e)** Relative power of the theta band. **(f)** Sample entropy of the theta band. **(g)** Relative power of the gamma band. **(h)** Sample entropy of the gamma band. *p < 0.05, **p < 0.01, ***p < 0.001.

Theta and gamma oscillations in the CA1 region are closely related to the working memory of mice, playing a crucial role in the encoding of spatiotemporal information and memory retrieval. During the NOR behavioral experiment, LFP signals in AD mice were recorded. Figures 2a–d show the typical LFP signals, corresponding power spectra and theta and gamma power spectra. We first analyzed the relative power of the theta band in the local field (Figure 2e). The results demonstrated that the theta wave relative power in the testing phase was significantly higher in both AD+PBM and health groups compared to the AD+Sham group (0.470 ± 0.032 and 0.449 ± 0.023 *vs.* 0.403 ± 0.030, *n* = 7, *p* < 0.05 and *p* < 0.01, respectively. We further analyzed the relative power of the gamma band in the LFP (Figure 2g). We found that the relative power of the gamma band in the AD+PBM group was significantly higher after PBM stimulation compared to the AD+Sham group but tended to be close to the health group (0.124 ± 0.016 *vs.* 0.102 ± 0.015 and 0.138 ± 0.018, *n* = 7, *p* < 0.01 and *p* < 0.05, respectively). At last, we analyzed the sample entropy of the theta and gamma bands in the LFP (Figures 2f, h). We found a significant increase in sample entropy in both theta and gamma bands in AD mice after PBM stimulation. In the theta band, the sample entropy in the AD+PBM group showed a significant increase compared to the AD+Sham group and the health group (0.1211 ± 0.0005 *vs.* 0.1224 ± 0.0013 and 0.1205 ± 0.0007, *n* = 7, *p* < 0.01, and *p* < 0.05, respectively). Similar results were obtained in the gamma band (0.567 ± 0.001 *vs.* 0.570 ± 0.001 and 0.565 ± 0.003, *n* = 7, *p* < 0.01, and *p* < 0.05, respectively). In summary, PBM stimulation for 62 days significantly increased the relative power and sample entropy of theta and gamma bands in AD mice. We also analyzed the relative power and sample entropy of theta and gamma bands in healthy mice after PBM. The relative power and sample entropy of theta band were (0.47 ± 0.032 vs. 0.51 ± 0.027, *p* < 0.05) (Figure 2e) and (0.12 ± 0.0007 vs. 0.12 ± 0.0006, respectively. *p* < 0.05) (Figure 2f). The relative power and sample entropy in gamma band were (0.14 ± 0.018 vs. 0.16 ± 0.013, *p* < 0.05) (Figure 2g) and (0.56 ± 0.003 vs. 0.56 ± 0.002, *p* < 0.05) (Figure 2h), respectively. These indicates that PBM can improve the non-specific cognitive function of mice.

### 3.3 PBM modulates phase-amplitude coupling strength in theta, delta, and gamma bands in the CA1 region of AD mice

**FIGURE 3:**
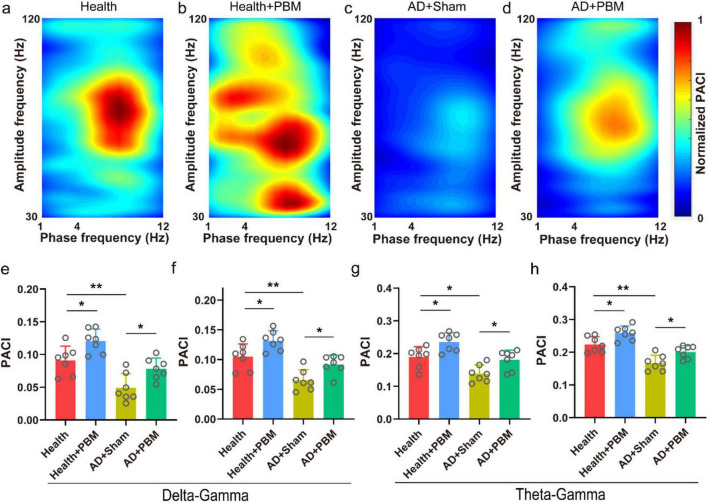
**(a)** Typical phase amplitude coupling intensity (PACI) map of Health group mice (randomly selected data for 1-min, non-average data). **(b)** Typical PACI map of Health+PBM group mice. **(c)** Typical PACI map of AD+Sham group mice. **(d)** Typical PACI map of AD+PBM group mice. **(e)** PACI results for delta-low gamma band. **(f)** PACI results for delta-high gamma band. **(g)** PACI results for theta-low gamma band. **(h)** PACI results for theta-high gamma band. *p < 0.05, **p < 0.01.

In addition to the aforementioned power spectrum analysis, we also calculated the PACI values for δ − low γ, δ − high γ, θ − low γ, and θ − high γ bands. This helps analyze changes in the collaborative capacity of the CA1 region with other brain areas in AD mice. Figures 3a–d show the typical phase-amplitude coupling strength maps for the health group, health+PBM group, AD+Sham group, and AD+PBM group mice, respectively. We observed that compared to the AD+Sham group mice, the coupling strength of δ − low γ in AD+PBM and health group mice was significantly stronger (Figure 3e, 0.078 ± 0.016 and 0.091 ± 0.022 *vs.* 0.049 ± 0.022, *n* = 7, *p* < 0.05, and *p* < 0.01, respectively). Additionally, the coupling strength of δ − high γ was also significantly stronger in AD+PBM and health group mice compared to the AD+Sham group (Figure 3f, 0.092 ± 0.016 and 0.105 ± 0.021 *vs.* 0.065 ± 0.018, *n* = 7, *p* < 0.05 and *p* < 0.01, respectively). Moreover, the coupling strength of θ − low γ in AD+PBM and Health group mice was significantly higher than in the AD+Sham group (Figure 3g, 0.181 ± 0.030 and 0.190 ± 0.031 *vs.* 0.139 ± 0.026, *n* = 7, *p* < 0.01 and *p* < 0.05, respectively). Similarly, the coupling strength of θ-high γ in AD+PBM and Health group mice was significantly stronger than in the AD+Sham group, as illustrated in Figure 3h (0.224 ± 0.024, 0.200 ± 0.019 vs. 0.167 ± 0.024, *N* = 7, *p < 0.05, **p < 0.01, Ordinary one-way ANOVA). Our results demonstrated that PBM stimulation for 62 days significantly enhanced working memory in AD mice, potentially influenced by the increased collaborative capacity of the CA1 region across brain areas. We also analyzed PACI in multiple frequency bands of healthy mice after PBM stimulation, and the results were as follows: delta-low gamma (0.091 ± 0.022 vs. 0.12 ± 0.018, *p* < 0.05) (Figure 3d), delta-high gamma (0.11 ± 0.021 vs. 0.13 ± 0.017, *p* < 0.05) (Figure 3f), theta-low gamma (0.19 ± 0.031 vs. 0.23 ± 0.028, *p* < 0.05) (Figure 3g) and theta-high gamma (0.22 ± 0.024 vs. 0.25 ± 0.022, *p* < 0.05) (Figure 3h). The above results demonstrated that PBM had significant effects on the PAC of CA1 region in both healthy and AD mice.

## 4 Discussion

In this study, continuous stimulation of 1070 nm near-infrared light at 10 Hz for 62 days significantly improved the working memory of AD model mice, demonstrating that PBM can enhance cognitive abilities in AD mice. Concurrently, the analysis of collected LFP signals revealed that 10 Hz PBM enhances the relative power and sample entropy in the theta and gamma bands during the execution of cognitive tasks and the phase-amplitude coupling strength of theta and delta bands with the gamma band. These findings align with the improvement in the mice’s memory capabilities, suggesting that PBM may enhance memory in AD mice by improving these neural oscillation features.

While the exact mechanism of PBM remains unclear, it is believed that near-infrared light can activate chromophores, including cytochrome c oxidase and heat-gated ion channels, and restore cellular function through multi-level mechanisms (Urquhart et al., 2020; Tian et al., 2016; Salehpour et al., 2018). PBM regulates the formation of reactive oxygen species and activates transcription factors that can upregulate or downregulate the expression of numerous genes, including nuclear factor kappa B. This factor regulates the expression of over 100 genes, including proteins with antioxidant, anti-apoptotic, pro-proliferative, and pro-migratory functions (Hamblin, 2018). The improvement in cellular function is likely to cause changes in neural oscillations. Thus, PBM may enhance memory in AD model mice by influencing cellular functions.

In addition to its indirect impact on cellular functions, PBM may also improve the neural oscillation features of the CA1 region by influencing brain network connections. Research has shown that PBM significantly enhances the alpha power of multiple resting-state networks in the brain and the hemodynamic activity in cortical areas. It strengthens functional connectivity between the frontal and parietal regions, which can significantly impact mice’s working memory (Zhao et al., 2022). Therefore, the effects of PBM on cellular function and brain network connections may be contributing factors to the changes in neural oscillations in the CA1 region.

Previous literatures have demonstrated that PBM can ameliorate the pathology associated with Alzheimer’s disease (AD) (Purushothuman et al., 2014; Grillo et al., 2013; Farfara et al., 2015). Our histological results as illustrated in Supplementary Figure 4 corroborated to the above conclusion. Previous investigations have indicated that alterations in gamma oscillations within AD mouse models are linked to changes in spatial memory, which align with pathological findings (Yao et al., 2020; Iaccarino et al., 2016). These pathological alterations may also underlie modifications in neural oscillation patterns.

We calculated DI and exploration time as shown in Supplementary Figure 5. The results demonstrated that the PACI of mice in the AD+PBM group during the Y maze test was significantly higher than that of unstimulated AD mice, which proved the reproducibility of our results.

To rule out the impact of surgery on LFP, we added new experiments. Six healthy mice were implanted with electrodes, and the relative powers of theta and gamma frequency bands of the LFP signal were analyzed at the time of electrode implantation, one day later, and two days later. We found no significant differences between them (shown in Supplementary Figure 6). The above results prove that surgery has no significant impact on LFP signal.

This study investigated changes in neural oscillation features of LFP in the CA1 region using a single electrode and did not analyze the fundamental reasons for LFP changes. Future research could employ multi-channel electrodes to analyze changes in different types of neurons in the CA1 region. Additionally, collecting electronic signals from multiple brain regions would facilitate analyzing changes in brain network connections. As a non-invasive, safe, and convenient novel brain modulation technique, PBM holds high application value and potential. This research provides a theoretical foundation for applying PBM to treating AD, contributing to its potential early clinical application.

## Data Availability

The original contributions presented in this study are included in this article/Supplementary material, further inquiries can be directed to the corresponding author.
