# Supplementary material for: Near-infrared light stimulation regulates neural oscillation and memory behavior of mice with Alzheimer’s disease
Source: Front Neurosci. 2024 Oct 21;18:1417178. doi: 10.3389/fnins.2024.1417178 (PMC11532060; doi:10.3389/fnins.2024.1417178)
Supplement: Supplementary file 1 [file Data_Sheet_1.docx]

**Near-Infrared Light Stimulation Regulates Neural Oscillation and Memory Behavior of Mice with Alzheimer's Disease**

**Song Zhang^1^, Xiaopeng Wang^1^*, Honglei Jiao^1^,**

1.Department of Neurology, The Second Hospital of Hebei Medical University, Shijiazhuang, China

***Correspondence:**

Xiaopeng Wang, MD. Ph.D.

Email: wang_xiaopeng@126.com


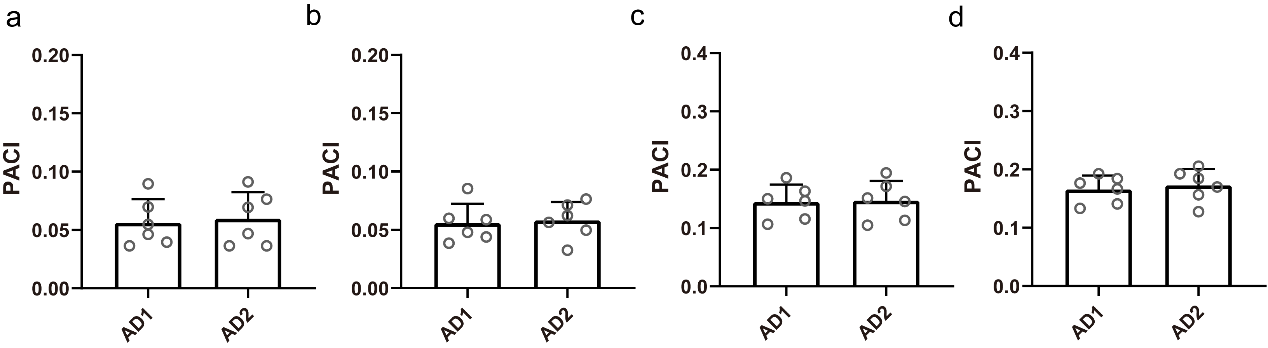


Figure. S1. AD mice were randomly selected and randomly divided into two groups: AD1 and AD2, there was no significant difference in PACI between two groups. (a-d) delta-low gamma, delta-high gamma, theta-low gamma and theta-high gamma.


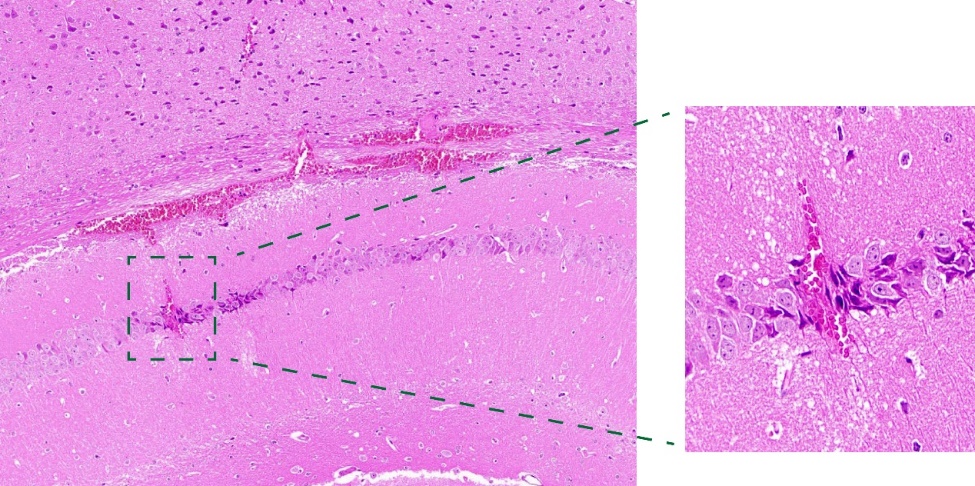


Figure. S2. HE stained section at electrode implantation location.


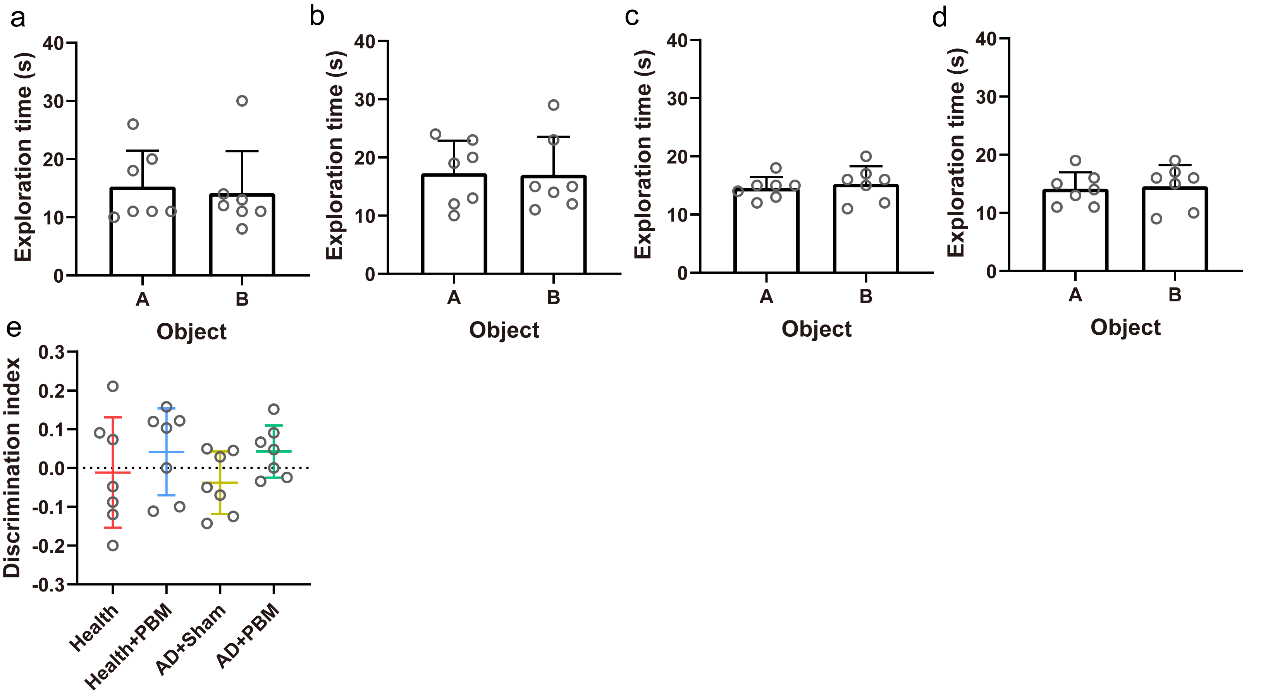


Figure. S3. The exploration time for mice to explore each object, (a) Health group, (b) Health+PBM group, (c) AD+Sham group, (d) AD+PBM group to explore each object in the learning stage, respectively. (e) The DI value of each group in the learning stage.


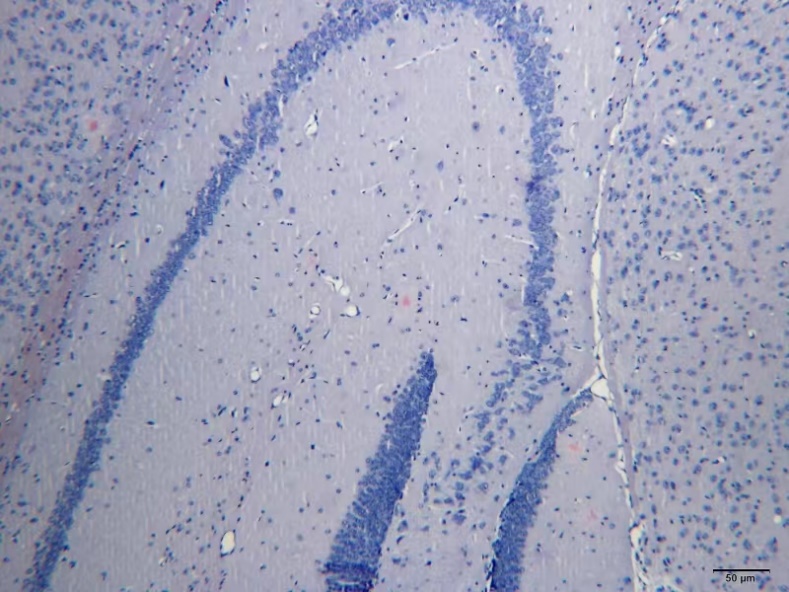


Figure. S4. Histological sections of the hippocampal region of AD mouse.


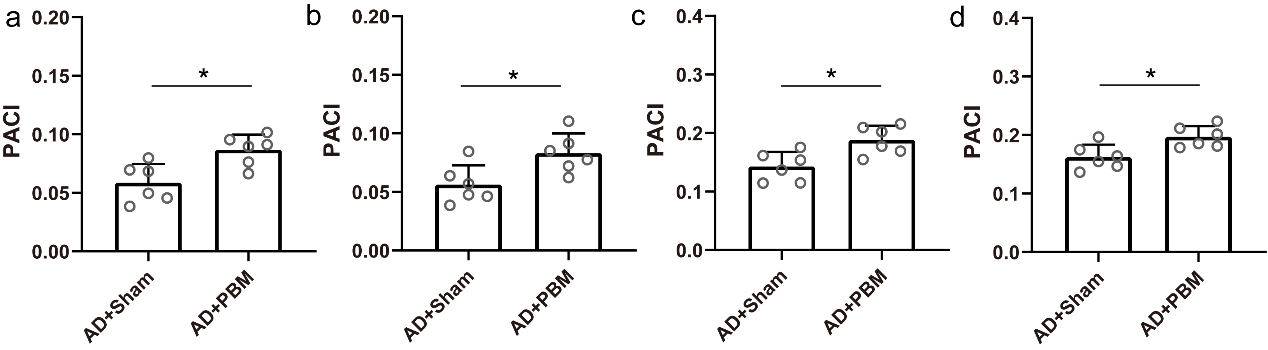


Figure S5. PACI of AD mice with PBM in Y-maze experiment


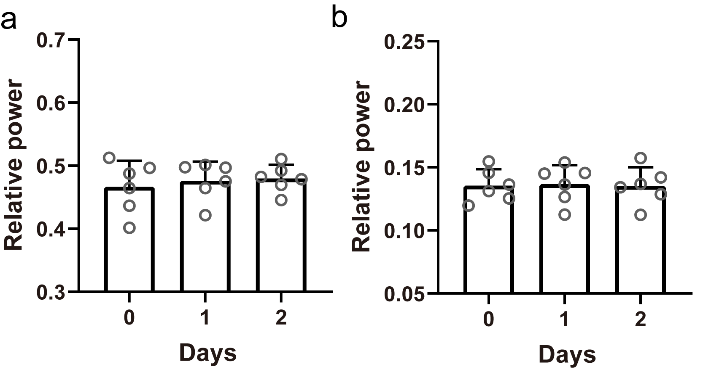


Figure.S6. The relative power of LFP signals in healthy mice on the 0,1 and 2 days after electrode implantation. (a) theta band (b) gamma bands.
